# Supplementary figures and images for: The corepressor NCOR1 and OCT4 facilitate early reprogramming by suppressing fibroblast gene expression
Source: PeerJ. 2020 Apr 22;8:e8952. doi: 10.7717/peerj.8952 (PMC7183309; doi:10.7717/peerj.8952)

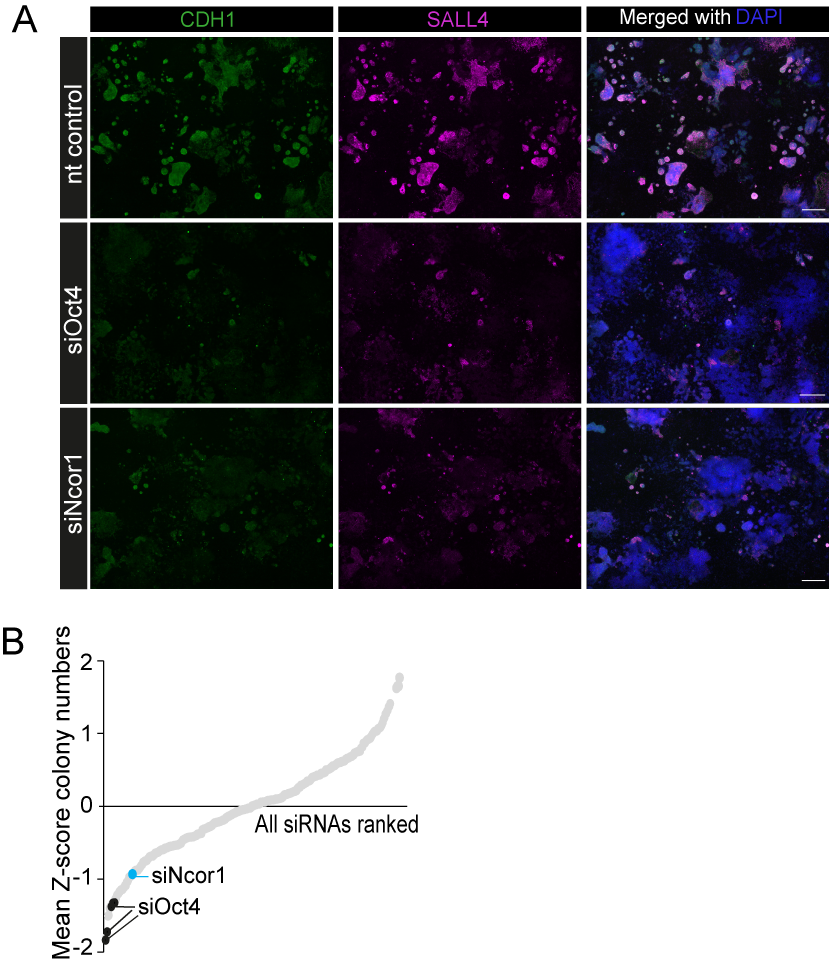

Supplement: Supplemental Information 1 — (A) High-content images from 9 stitched-fields spanning the whole 96-well, for Ncor1, Oct4 or nt control siRNAs. In green CDH1 staining, SALL4 in magenta and the overlay with DAPI nuclear counterstain. The scale bar represents 500 uM. (B) Derived from the HC-screen (Peñalosa-Ruiz et al., 2019), reprogramming efficiencies (colony numbers) from + 300 siRNAs, ranked by their Z-score average of four replicates. The black labels correspond to siOct4 and siNcor1 (blue) also scores low in colony numbers. [file peerj-08-8952-s001.png]

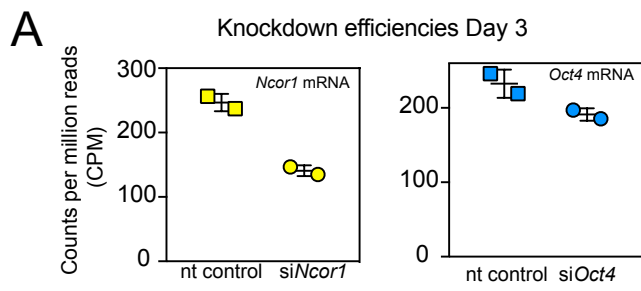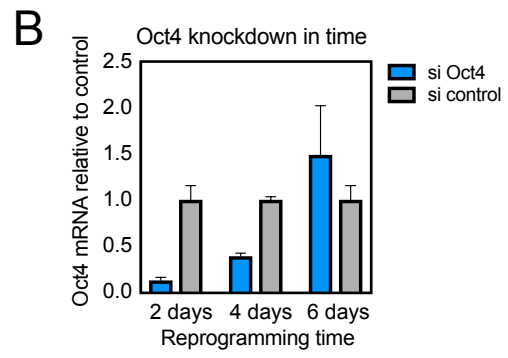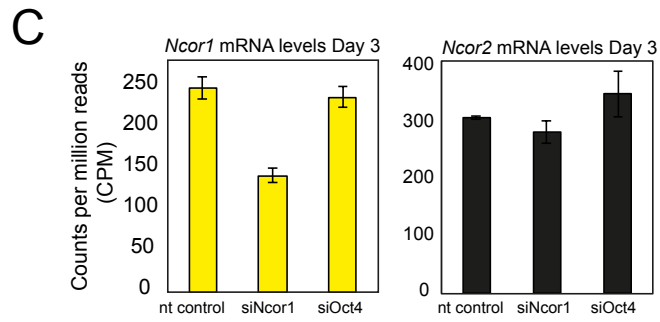

Supplement: Supplemental Information 2 — (A) Transcript counts per million reads (CPM) for Ncor1 (yellow) and Oct4 (blue) siRNAs, compared to nt control after RNA-seq, show that the corresponding mRNAs were targeted. (B) Time-course for Oct4 mRNA silencing at different reprogramming time-points. Data was derived from RT-qPCR, where Oct4 mRNA levels were determined relative to tubulin according to the ∆∆Ct method. Then, expression values in nt control were set to 1 and Oct4 remaining expression levels in siOct4 (blue) were calculated relative to nt values, per time-point. The bars represent the average of two independent transfection replicates +/− Standard Deviations. (C) Ncor1 mRNA expression (yellow) measured from RNA-seq data at reprogramming Day 3 in non-targeting control siRNA (nt control, Bakerersfield, CA, USA), siNcor1 and siOct4 (left). Ncor1 mRNA expression (black) measured from RNA-seq data at reprogramming Day 3 in non-targeting control siRNA (nt contro, Bakerersfield, CA, USA), siNcor1 and siOct4 (right). [file peerj-08-8952-s002.pdf]

A

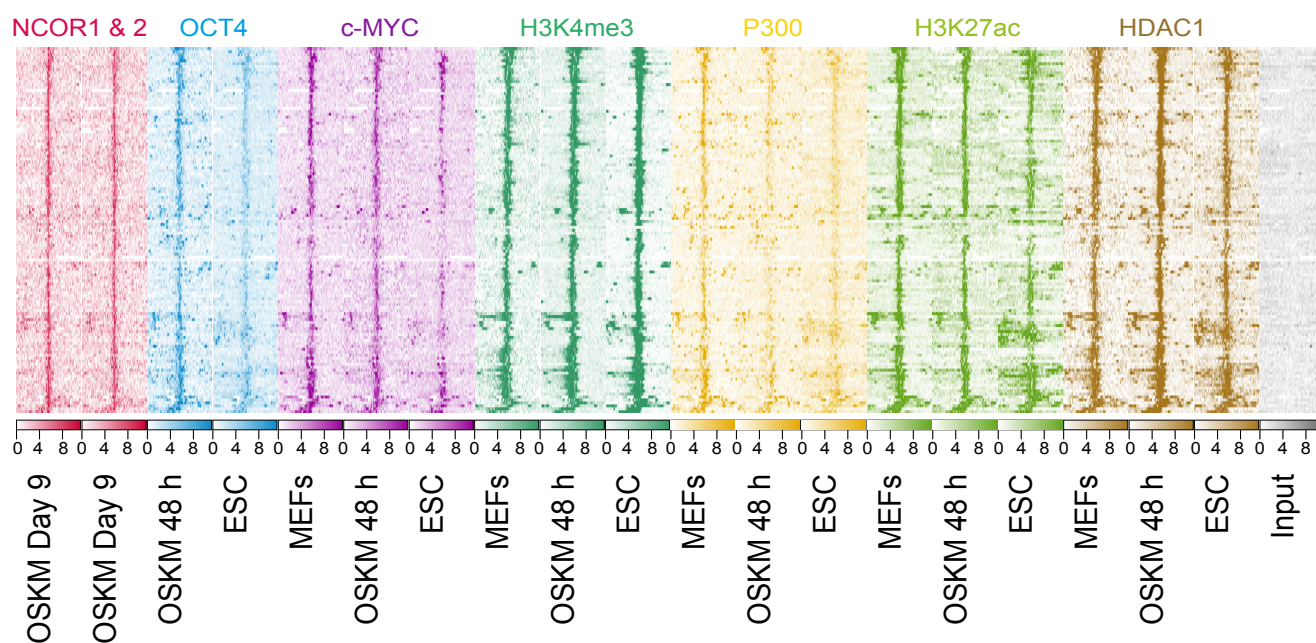

B

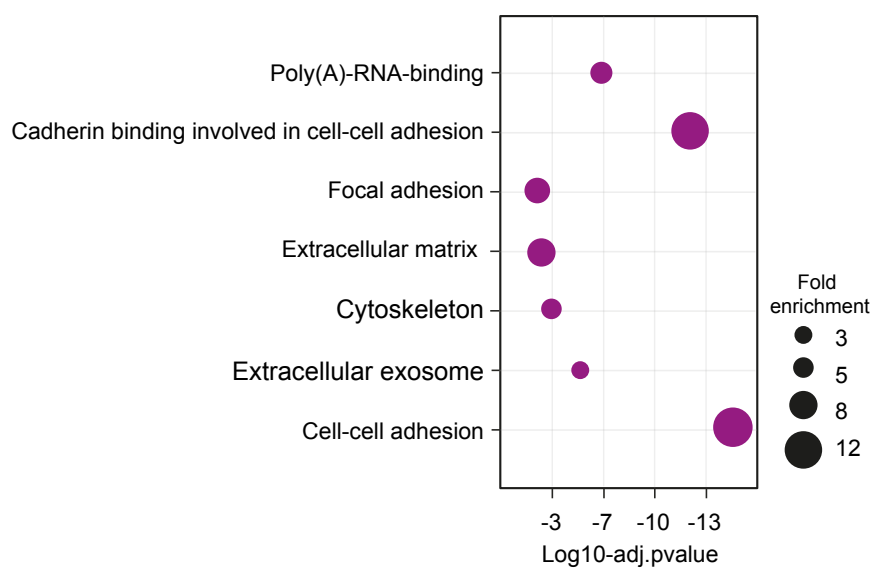

Supplement: Supplemental Information 3 — (A) Gene set analysed in this heatmap (vertical) corresponds to upregulated genes in siNcor1 (our RNA-seq data), which are also bound by NCOR1 protein by ChIP in Zhuang´s data (intersection of 121 genes in Fig. 4C, left). The heatmap represents ChIP-seq enrichment of NCOR1, NCOR2, OCT4, c-MYC proteins. We have also included histone modifications H3K4me3, H3K27ac, binding of acetyltransferase P300 and HDAC1 histone deacetylase [10, 22] to such genomic regions. The heatmap shows ChIP signals (color intensity) at genomic locations representing peak summits (center) plus and minus 5 kb (left-right). Vertically different genomic locations are shown. Scales represent RPKM-normalized ChIP-seq signals. (B) Bubble chart depicting GO classification of genes upon Ncor1 knockdown and bound by NCOR1 from ChIP-seq data. The size of the bubble represents the fold enrichment and the x-axis the log10 of the adjusted p-value.This analysis was performed with genes corresponding to Fig. 4D. [file peerj-08-8952-s003.pdf]
